# The Surgical Treatment of Obesity

**Published:** 1913-03-29

**Authors:** 


### THE SURGICAL TREATMENT OF OBESITY.

Every now and then some adventurous surgeon undertakes the relief of extreme obesity by operation. A San Francisco surgeon has recently published details of a lipectomy, which is interesting as showing the possibilities of modern surgery.

The patient was a lady of forty-two years of age, who weighed nineteen stone, although she stood but five feet two and a half inches in height. She had had eight children, and since the birth of the last one had suffered from an umbilical hernia. Her father weighed fifteen stone and her mother over fourteen. Having been advised to submit to operation for the relief of the hernia, she took the opportunity at the same time to be relieved of some of her superfluous fat. By the operation of lipectomy the surgeon removed a piece of skin and underlying fat one yard and three inches in length, eighteen inches wide, and three inches thick; this mass weighed seventeen pounds, a mere trifle compared with the weight of the enormously fat patient from whom it was removed. However, the surgeon supervised her diet as well, with the result that when after five weeks she was allowed to return to her home, her weight had diminished by seventy-five pounds to the still respectable figure of thirteen stone eight. By continuing a very carefully arranged diet it is hoped to reduce her eventually to ten stone or so.

The operative procedure requires careful attention to detail. To get a good result in these cases of extreme corpulence it is necessary to commence the incision well round at the back of the patient, and this renders the aseptic technique (on which all else depends) liable to failure unless it is thoughtfully planned out beforehand. An incision beginning two inches to one side of the spine is carried forwards across the abdomen above the umbilicus to a corresponding point on the other side. Another incision begins and ends at the same two points, but is carried much lower across the abdomen; in fact, just above the symphysis pubis. The flap above the upper incision and that below the lower one are undermined for about an inch, so as to allow of easier closing of the wound afterwards; and then the isolated mass outlined by the incisions is dissected right down to the deep fascia and removed in one piece. The one point to be careful of is the umbilicus, where a hernia may exist as in the San Francisco case. The enormous wound is carefully closed by three separate sets of sutures; a row of silk-worm gut tension sutures which take up everything in the wound, including the deep fascia, and are tied through fine rubber tubes to prevent them from cutting through; a continuous buried suture for the superficial fascia and fat; and a skin suture of any sort that the operator prefers, provided that the neatest and most exact apposition of the skin edges is secured. A light bandage to the abdomen, and a form of back rest which slightly flexes the spine so as to remove tension from the suture line, are the main requisites.

